# A Strychnia Eater, Etc.

**Published:** 1875-05

**Authors:** 


### A STRYCHNIA EATER—STRYCHNIA AN ANTIDOTE TO ALCOHOL.

The newspapers contain an account of a man residing at Gilroy, Cal., who is in the habit of using strychnia in enormous quantities to counteract the effects of drunkenness. Not wishing to give currency to a statement on mere newspaper authority, we wrote to Dr. H. C. Morey, of Gilroy, whose name was mentioned in connection with the matter, asking him to send us an authentic report of the facts of the case. In answer to our request, Dr. Morey kindly furnishes the following statement.—[Ed. *P. M. & S. Journal.*]

Gilroy, March 15, 1875.

Your letter of inquiry in reference to my personal knowledge of the reported strychnia eater, is at hand, and I hasten to reply. The individual in question is a man about fifty-two years of age, about five feet eight inches in height, and weighs about one hundred and fifty-eight pounds ; dark complexion, very plain in appearance, very eccentric and peculiar in his habits, and always keeps his own counsel ; has a good intellect, but a limited education.

I first became acquainted with this man in the fall of 1861, and soon learned of his habit of eating strychnia, after a long and continued debauch, and in a condition bordering on delirium tremens. The first time my attention was particularly called to it, he wished me to give him a bottle of strychnia, which I did at night about bed time. He took the bottle, pouring the strychnia into his hand, and threw it into his mouth as carelessly as though it were salt ; and, in the course of half an hour, not feeling the effects from it that he wished, he repeated it, and continued to do so until he became perfectly sober. The quantity required would correspond to the length of time he had been drinking, and the quantity of whisky he had drank. I was struck with the wonderful effect it had to so completely sober him, and leave his system so entirely free of any nervous disturbance, and without the reddened and bloated appearance of the face, the dull, heavy eyes, and irritable stomach of the drunkard. After a two weeks' drunk, with all the appearances of approaching delirium tremens, he got up in the morning with his mind clear, his eyes bright, his skin clear and fair, and with all the appearances of a man in perfect health and vigor, and ate as hearty a breakfast as usual, and went to his work as though he had never taken a drop of whisky in his life. My curiosity being excited at what seemed so unaccountable an occurrence, I began questioning him as to when he commenced its use, and what induced him to take it, but found him very reticent, and have not, to this day, ascertained the causes that first led him to its use. All he will tell is that he commenced its use in 1856. From 1861 to 1867, I saw him very frequently, and almost as often have seen him take the strychnia, until it ceased to be a curiosity, except to study its physiological action. In every instance when he took it, every appearance of dissipation would disappear in a very short time. Whether *strychnia* is an antidote to alcoholic poison, and *vice versa*, was a study for which I could find no authority to guide my conclusions.

From 1867, I did not see him until the month of November, 1874, when he came to this place and called on me for strychnia, as of old. I told my clerk (Mr. Martin) to give him all he wanted. He gave him a bottle, from which he took about twenty grains. In an hour he was all right, and sober as ever. This caused Mr. Martin to write the article you can find in the "Druggists' Circular" of December, 1874, and from that article arose the newspaper articles.

I have experimented with strychnia and *nux vomica* as an antidote to the effects of alcohol, and invariably with beneficial results.

